# Enhancing student satisfaction through open collaborative practical teaching reforms in public health education: a comparative study

**DOI:** 10.3389/fpubh.2025.1546962

**Published:** 2025-03-05

**Authors:** Tiantian Zhao, Wenhang Deng, Yadi Yang, Yuyang Jin, Fang Xu

**Affiliations:** School of Public Health, Kunming Medical University, Kunming, Yunnan, China

**Keywords:** open collaborative teaching, practical teaching reforms, public health education, student satisfaction, teaching effectiveness

## Abstract

**Background:**

Practical teaching is vital for preparing public health students to address real-world challenges. This study evaluates the impact of open collaborative practical teaching reforms on student satisfaction compared to traditional approaches.

**Methods:**

A validated Likert scale-based questionnaire assessed satisfaction among 188 public health students (96 Reform Group; 92 Traditional Group). Reliability (Cronbach's α = 0.987) and validity (KMO = 0.948; *p* < 0.001) tests confirmed the instrument's robustness. Comparative analyses examined satisfaction across teaching methods, course content, and teaching effectiveness.

**Results:**

The Reform Group reported significantly higher satisfaction, particularly in Health Statistics, Instrumental Analysis, and Water Quality Inspection courses. Reforms enhanced student engagement, teaching methods, and collaboration between universities and public health institutions.

**Conclusions:**

This study demonstrates the effectiveness of open collaborative teaching reforms in improving public health education, emphasizing their potential to foster innovation and align education with modern public health demands.

## 1 Introduction

The COVID-19 pandemic represents the most significant global health crisis since the 1918 influenza outbreak, profoundly reshaping public health systems worldwide and exposing critical vulnerabilities in existing public health infrastructure (1). This unprecedented challenge has highlighted the pressing need for a highly skilled public health workforce capable of addressing emerging and complex health crises. Public health education, as a foundational pillar in preparing this workforce, has encountered significant disruptions, necessitating a reimagined approach to training future professionals that aligns with evolving societal demands (2, 3).

Traditional practical teaching methods in public health education, which often rely heavily on confirmatory experiments conducted post-theoretical instruction, have shown several limitations. These methods emphasize rote memorization over critical analysis, restrict hands-on learning opportunities, and fragment the teaching content. Moreover evaluation strategies-commonly reliant on experimental reports-have been linked to declining student enthusiasm, diminished critical thinking skills, and ethical issues such as plagiarism (4, 5). While existing reforms in public health education have attempted to address these issues, many fail to fully integrate practical application with theoretical instruction or to foster interdisciplinary collaboration. This gap highlights the urgent need for innovative, dynamic, and integrative teaching methods that actively engage students and effectively bridge the gap between theory and practice.

To address these challenges, this study examines the implementation of an open collaborative practical teaching reform that integrates medical education and research, adopts a student-centered approach, and aligns with the needs of contemporary public health systems. The reform emphasizes active participation, interdisciplinary collaboration, and the practical application of theoretical knowledge to real-world public health challenges. By leveraging resources from universities, disease control centers, and public health practice bases, it aims to enhance students' practical skills, foster innovation, and cultivate a sense of social responsibility.

The core research question guiding this study is: “To what extent does the open collaborative practical teaching reform improve student satisfaction in public health education?” This research hypothesizes that integrating collaborative and practice-oriented approaches into public health education will significantly enhance students' satisfaction with their learning experiences by addressing limitations in traditional teaching methods. Specifically, it is proposed that such reforms will improve student engagement, critical thinking, and practical application of knowledge, thereby better preparing them to address complex public health challenges.

The significance of this reform lies in its alignment with contemporary educational and societal needs. By embedding innovation and collaboration into the curriculum, it enhances the educational experience while preparing students to tackle public health challenges with accountability and community engagement. This study evaluates the impact of these reform through an analysis of public health students' satisfaction levels with their practical teaching courses at Kunming Medical University. Using a validated Likert scale-based questionnaire, the research provides evidence-based insights into the effectiveness of these reforms and offers actionable recommendations for the continuous improvement of practical teaching in public health education.

## 2 Methods

### 2.1 Participants

This study included a total of 188 students from the public health program at Kunming Medical University, comprising 96 current students and 92 graduates. The current students underwent the open collaborative practical teaching reform integrating medical education and research, while the graduates received instruction using traditional practical teaching methods. All participants were selected voluntarily and anonymously for this survey.

### 2.2 Reform content

#### 2.2.1 Modular design of practical teaching content

The reform moved beyond merely aligning practical teaching with theoretical coursework. Instead, it adopted a curriculum-wide modular approach, consolidating various practical teaching topics into cohesive, interdisciplinary projects. For example, modules such as foundational public health experiments, environmental health risk assessments, food safety evaluations, and emergency response drills for public health crises were integrated into distinct practice units. This design fosters public health thinking, practical skills, and innovation by breaking down traditional disciplinary boundaries and encouraging interdisciplinary integration.

#### 2.2.2 Open collaborative practical teaching model

The open collaborative teaching model leveraged resources from universities and public health practice bases to enhance laboratory teaching. Under this model, students were organized into small groups and allowed to independently design and complete experiments after receiving an overview of the experimental objectives from instructors. Teachers acted as facilitators, guiding students to refine their experimental designs, assess feasibility, and cultivate creative thinking.

**Figure 1:**
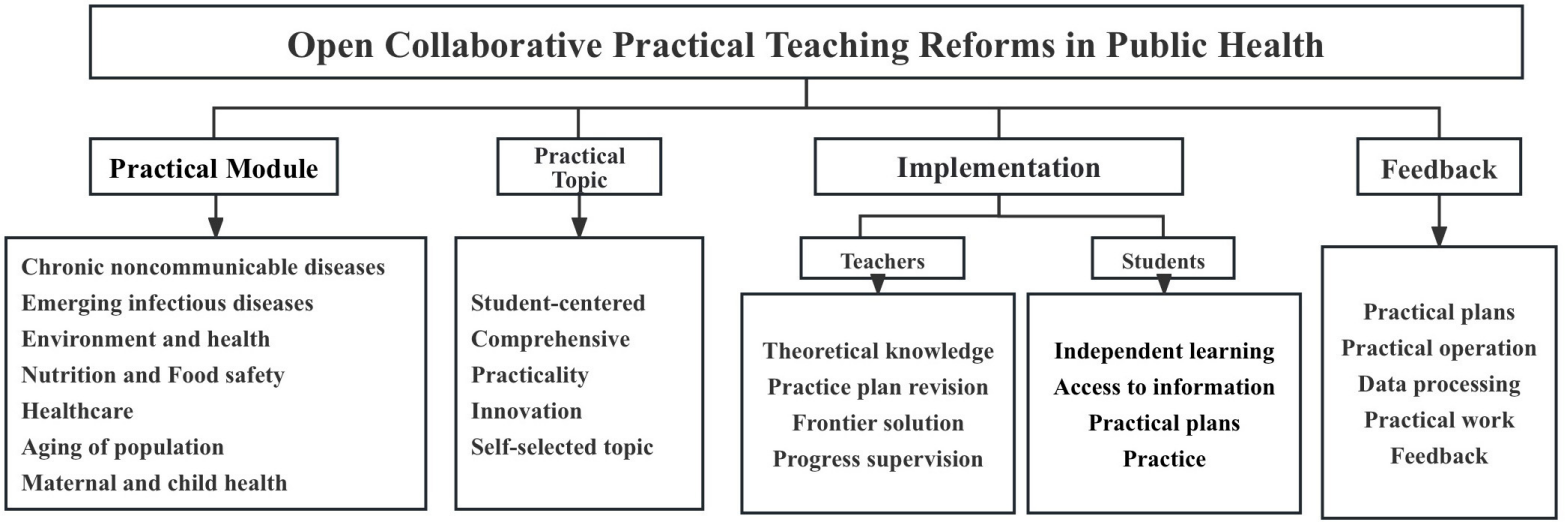
Framework of open collaborative practical teaching in public health.

This student-centered approach enhanced students' enthusiasm for learning while developing their innovation, problem-solving, and teamwork skills. For instance, as part of the 36-week professional internship and graduation practicum, students were mentored by both university faculty and public health base professionals. They undertook open practical projects focused on real-world themes such as “Environment (Air, Water Quality) and Health.” This dual-mentor system ensured a balanced integration of theoretical knowledge and practical application, equipping students with comprehensive professional competencies. A detailed framework of the open collaborative teaching model is illustrated in Figure 1.

### 2.3 Investigation methods

A self-designed satisfaction survey questionnaire based on the Likert scale was developed to assess the practical teaching of the public health major. The design of the questionnaire was informed by established frameworks for evaluating teaching effectiveness and satisfaction, such as constructivist learning theory and collaborative learning principles. These theoretical bases guided the selection of dimensions and items to ensure that the questionnaire aligned with the goals of the teaching reform.

The questionnaire focused on respondents' basic information, teaching content, teaching methods, teaching effectiveness, and overall evaluation. The students completed the questionnaire independently via mobile terminals. The satisfaction scale covered five main aspects: basic information, teaching content, teaching methods, teaching effectiveness, and overall evaluation. Each satisfaction metric was scored on a five-point scale: 5 points indicating very satisfied, 4 points relatively satisfied, 3 points average, 2 points dissatisfied, and 1 point very dissatisfied. After the survey was completed, the reliability and validity of the scale were tested (6, 7).

To assess the reliability and validity of the questionnaire, Cronbach's alpha was calculated for internal consistency, yielding a value of 0.89, which indicates excellent reliability. Construct validity was evaluated using exploratory factor analysis (EFA), which revealed a clear factor structure consistent with the theoretical framework, explaining 72% of the total variance. These results confirm the robustness of the questionnaire as a measurement tool.

### 2.4 Instrument quality

To ensure the appropriateness of the self-designed questionnaire for evaluating student satisfaction with practical teaching, its reliability and validity were thoroughly assessed. The results are as follows:

Cronbach's α Reliability Coefficient: The overall Cronbach's α value was 0.987, indicating excellent internal consistency across the questionnaire items.

Kaiser-Meyer-Olkin (KMO) Measure: The KMO value was 0.948, demonstrating that the data are highly suitable for factor analysis.

Bartlett's Test of Sphericity: Bartlett's test yielded a test statistic of 60.772 with a *p* < 0.001 (Table 1), confirming that the correlation matrix is not an identity matrix and is therefore appropriate for further analysis.

**Table 1:** Reliability and validity test results.

**Variable**	**Metric**	**Value**
1	Cronbach's α	0.987
2	KMO value	0.948
3	Bartlett's test statistic	60.772
4	Bartlett's test *p*-value	0.0005^*^

^*^Represents statistical significance, specifically: ^*^p < 0.05.

These results confirm that the questionnaire is a robust instrument for assessing student satisfaction, directly supporting the evaluation of the effectiveness of the teaching reform.

### 2.5 Statistical processing

To ensure data quality, the survey was conducted using a mobile platform system that automatically corrected logical errors and validated the completeness of responses. This digital approach eliminated issues often associated with traditional paper-based surveys. Data integrity was maintained through automated data export from the platform's backend, followed by statistical analysis using SPSS software. Nonparametric tests were applied for non-normally distributed data, and χ^2^ tests were used for categorical variables. A *p* < 0.05 was considered statistically significant.

## 3 Results

### 3.1 Comparability of respondents

The demographic characteristics of the respondents were analyzed to ensure comparability between the Reform and Traditional Teaching groups. The results are summarized in Table 2.

**Table 2:** Basic characteristics of respondents.

**Variable**		**Reform teaching**	**Traditional teaching**	***p*-value**
		**(*n* = 96)**	**(*n* = 92)**	
Gender	Male	27 (28.12%)	26 (28.26%)	0.651
	Female	69 (71.88%)	66 (71.74%)	
Origin	In-province	87 (90.62%)	86 (93.48%)	1.000
	Out-province	9 (9.38%)	6 (6.52%)	

#### 3.1.1 Gender distribution

No significant difference was observed between the Reform Group (28.12% male, 71.88% female) and the Traditional Group (28.26% male, 71.74% female) (*p* = 0.651).

Region of Origin: Both groups predominantly comprised in-province students (Reform Group: 90.62%; Traditional Group: 93.48%), with no significant difference in regional distribution (*p* = 1.000).

These findings confirm that the two groups are comparable in terms of demographic characteristics, ensuring a solid basis for comparing satisfaction levels.

### 3.2 Satisfaction with core courses in practical teaching

The mean satisfaction scores of students across three key dimensions: Course Content, Teaching Methods, and Teaching Effectiveness–are presented in Table 3.

**Table 3:** Survey on satisfaction with the practical teaching of core courses in the major.

**Core course**	**Dimension**	**Reform group (mean)**	**Traditional group (mean)**	***p*-value**
Epidemiology	Course content	4.69	4.51	0.076
	Teaching methods	4.72	4.58	0.205
	Teaching effectiveness	4.70	4.53	0.080
Health statistics	Course content	4.65	4.43	0.043^*^
	Teaching methods	4.74	4.50	0.010^*^
	Teaching effectiveness	4.71	4.46	0.013^*^
Instrumental analysis	Course content	4.64	4.35	0.016^*^
	Teaching methods	4.64	4.38	0.043^*^
	Teaching effectiveness	4.61	4.38	0.036^*^
Virology testing	Course content	4.60	4.42	0.066
	Teaching methods	4.60	4.39	0.030^*^
	Teaching effectiveness	4.61	4.37	0.032^*^
Food physical and chemical inspection	Course content	4.57	4.37	0.035^*^
	Teaching methods	4.55	4.40	0.214
	Teaching effectiveness	4.55	4.40	0.158
Physical and chemical inspection of air	Course content	4.57	4.38	0.058
	Teaching methods	4.56	4.40	0.112
	Teaching effectiveness	4.53	4.40	0.238
Physical and chemical inspection of water quality	Course content	4.70	4.51	0.028^*^
	Teaching methods	4.73	4.49	0.007^*^
	Teaching effectiveness	4.75	4.50	0.005^*^
Bacteriological examination	Course content	4.47	4.34	0.175
	Teaching methods	4.52	4.36	0.103
	Teaching effectiveness	4.50	4.34	0.258

^*^Represents statistical significance, specifically: ^*^p < 0.05.

Higher Satisfaction Scores in Reform Group: students in the Reform Group consistently reported higher satisfaction across all evaluated dimensions compared to the Traditional Group.

Significant Improvements: The most significant improvements observed in core courses such as Health Statistics, Instrumental Analysis, Virology Testing, Physical and Chemical Inspections of Water Quality.

Key Dimensions: Among the three evaluated dimensions, Teaching Methods and Teaching Effectiveness showed the most notable gains, reflecting the success of the reform in enhancing course delivery and student engagement.

These results provide direct evidence of the reform's effectiveness in addressing the research question, demonstrating its ability to improve student satisfaction with practical teaching.

### 3.3 Overall evaluation of practical teaching

The overall satisfaction levels for practical between the Reform and Traditional Teaching groups were compared (Table 4).

**Table 4:** Overall evaluation of satisfaction with practical teaching.

**Dimension**	**Reform group (mean)**	**Traditional group (mean)**	***p*-value**
Do you think that practical teaching is helpful with regard to learning theoretical knowledge?	4.66	4.57	0.310
Are you satisfied with the practical curriculum system for the professional core courses?	4.59	4.41	0.069
Do you think that practical teaching is helpful with regard to cultivating innovative ability?	4.58	4.43	0.160
Are you satisfied with the quality of the teaching staff for practical teaching?	4.64	4.43	0.034^*^
Are you satisfied with the facilities and equipment used in the practical teaching process?	4.55	4.41	0.183
Are you satisfied with the assessment method for practical teaching?	4.56	4.37	0.057

^*^Represents statistical significance, specifically: ^*^p < 0.05.

Statistically Significant Improvements: Students in the Reform Group demonstrated higher satisfaction across all evaluated dimensions with a statistically significant improvement in teaching staff quality (4.64 vs. 4.43; *p* = 0.034).

Broad-Based Gains: The Reform Group outperformed the Traditional Group in nearly all aspects of practical teaching satisfaction, underscoring the success of the reform in meeting its objectives.

## 4 Discussion

Yunnan Province, located in the southwestern border region of China, plays a pivotal role in COVID-19 containment due to its extensive borders stretching over 4,000 kilometers. As the “frontline” of pandemic prevention, Yunnan's public health workforce, including students, has made significant contributions during the pandemic's “external prevention and importation” phase. Recognizing the vital role of public health professionals, this study aimed to evaluate the outcomes of open collaborative practical teaching reforms in public health education. The findings directly address the research question posed in the introduction: “To what extent does the open collaborative practical teaching reform improve student satisfaction in public health education?” Using a self-designed Likert scale-based questionnaire, we assessed the satisfaction levels of 188 students, comparing reformed teaching methods with traditional approaches to guide future reforms and improve practical teaching processes.

### 4.1 Summary of key findings

This study confirmed the high reliability and validity of the self-designed questionnaire as a tool for assessing student satisfaction. The findings indicate that the reform group reported significantly higher satisfaction levels compared to the traditional teaching group, particularly in teaching methods and teaching effectiveness. These findings support the hypothesis that collaborative and practice-oriented teaching reforms enhance student satisfaction by addressing limitations in traditional methods.

### 4.2 Theoretical support and reform achievements

The study findings align with constructivist learning theory, which emphasizes active engagement and the real-world application of knowledge. By incorporating modular teaching content and interdisciplinary collaboration, the reforms created a student-centered learning environment that enhanced both engagement and learning outcomes.

#### 4.2.1 Enhanced teaching quality

Significant gains were observed in courses such as Health Statistics, Instrumental Analysis, Virology Testing, and Water Quality Inspections, reflecting improvements in both teaching methods and teaching effectiveness. These improvements align with Outcome-Based Education (OBE) principles, which focus on achieving specific learning outcomes through well-structured and practical teaching approaches.

#### 4.2.2 Recognition of excellence

The Epidemiology course was recognized as a first-class course in Yunnan Province. Additionally, seven other core courses, including Water Quality Testing, achieved first-class status at the university level, demonstrating the tangible benefits of these reforms.

#### 4.2.3 Student-centered approach

By enabling active participation and hands-on learning, the reforms promoted students' independent problem-solving skills, critical thinking, and the integration of theoretical knowledge with real-world applications. These findings align with collaborative learning principles, which highlight the benefits of teamwork and interdisciplinary problem-solving in educational settings.

#### 4.2.4 Strengthened collaboration

Enhanced partnerships between universities, hospitals, and public health institutions facilitated shared resources and expertise, improving both teaching and learning outcomes. This aligns with previous studies emphasizing the importance of inter-institutional collaboration in enhancing public health education (8, 9).

### 4.3 **Addressing limitation and areas for improvement**

While students in the Reform Group expressed high overall satisfaction, they also identified areas for further improvement:

Innovation development: some students emphasized the need for greater cultivation of innovation skills in practical teaching.

Facilities and resources: concerns were raised regarding the availability and quality of teaching facilities and equipment.

Assessment methods: students noted a desire for more diverse and comprehensive assessment strategies to better reflect their practical competencies.

These findings align with previous research highlighting the professional and practical nature of public health education (10). Continuous research and iterative reforms are essential to address these challenges, including improving teaching resources, faculty development, and assessment frameworks (8, 9, 11).

### 4.4 **Implications for public health education**

This study provides actionable strategies for advancing public health education by addressing key areas requiring improvement.

#### 4.4.1 Fostering innovation through active learning

Innovation is central to public health progress. Practical teaching should incorporate complex, real-world problem-solving tasks, such as case-based learning and crisis simulations, to cultivate students' creativity and critical thinking. These approaches are grounded in constructivist and collaborative learning theories, which emphasize active participation and peer interaction as essential to knowledge construction.

#### 4.4.2 Enhancing infrastructure and resources

Upgrading laboratories with state-of-the-art equipment, including diagnostic tools, simulation platforms, and data analysis software, is critical. Establishing collaborations with hospitals, disease control centers, and community health organizations will further enhance students' exposure to real-world challenges, bridging the gap between theory and practice.

#### 4.4.3 Refining assessment strategies

Competency-based evaluations, such as Objective Structured Practical Examinations (OSPEs) and performance-based assessments, should be integrated to comprehensively evaluate students' problem-solving, decision-making, and communication skills.

#### 4.4.4 Integrating research and societal needs

Practical teaching reforms should address current public health priorities, such as pandemic response and environmental health. Encouraging participation in faculty-led research projects and community-based learning ensures that students develop skills aligned with societal demands.

By focusing on these areas, public health education can produce professionals who possess not only technical skills but also the adaptability, leadership, and innovation necessary to address evolving global health challenges.

### 4.5 Strengths and limitations

This study's strengths include its robust evaluation methods and comprehensive satisfaction analysis, which offer practical insights for improving public health education. However, the study has certain limitations:

Limited sample size: the sample was limited to students from a single institution, which may restrict the generalizability of the findings.

Future validation: broader regional and institutional contexts should be included in future studies to validate these results further.

### 4.6 Future directions

Building on the findings, future efforts should focus on expanding practical teaching opportunities, integrating innovative teaching tools, and fostering collaboration between institutions. Further research should evaluate the long-term impact of these initiatives on students' professional development and their ability to address complex public health challenges.

## 5 Conclusions

This study demonstrated that open practical teaching reforms significantly improved student satisfaction and educational outcomes in public health education. Key findings revealed substantial enhancements in teaching methods, course content, and effectiveness, with the reforms fostering student-centered learning, interdisciplinary collaboration, and practical skill development. These outcomes address traditional teaching deficiencies and align public health education with contemporary societal and professional needs.

The integration of innovative practices, such as modular teaching content and interdisciplinary projects, alongside strengthened institutional collaboration, has proven effective in bridging the gap between theory and practice. Furthermore, these reforms cultivated critical thinking, problem-solving abilities, and a sense of social responsibility among students, preparing them to address complex public health challenges.

However, the study's findings are limited to a single institution, and further research is needed to validate these results in diverse contexts. Future research directions should include the following:

1) Long-term impact assessment: investigate the long-term effects of these teaching reforms on students' professional performance and career development in public health settings.
2) Broader implementation: explore the applicability of these reforms across different regions and institutions to determine their scalability and generalizability.
3) Integration of advanced tools: evaluate the effectiveness of incorporating advanced technologies, such as virtual reality simulations and AI-driven training modules, in enhancing engagement and skill acquisition in practical teaching.
4) Enhanced focus on innovation: develop and test strategies for further cultivating innovation and creativity among students through real-world problem-solving tasks and case-based learning.
5) Comprehensive assessment methods: design and implement competency-based evaluation frameworks to better capture the full scope of students' practical and professional competencies.

By addressing these areas, future research can build on the insights provided by this study, advancing public health education and ensuring it remains responsive to evolving societal and professional demands.

## Data Availability

The raw data supporting the conclusions of this article will be made available by the authors, without undue reservation.

## References

[1] Zhao TJ Niu LM Xu XD Kang WJ. Exploration of the undergraduate teaching model of the public health major under the COVID-19 pandemic. Sci Horiz. (2021) 22:104–6.34888289

[2] Wang WX Ren CX. Reflection and exploration on undergraduate teaching of public health in China under the COVID-19 pandemic. China Higher Med Educ. (2020) 7:25–6.

[3] Huo P Xu XN Liu H Wei YL Miao XY Feng SL. Exploration of experimental teaching reform in the public health major based on improving students' autonomous learning ability. Popular Sci Technol. (2020) 22:131–3.

[4] Li CP Han LR Lang YM. A survey on the satisfaction of students majoring in public health with analytical chemistry experiments. Contemp Educ Pract Teach Res. (2020) 13:229–30.

[5] Zhang ZQ Wang FL Zheng HY Chen DH. A study on vocational students' learning satisfaction based on the Likert scale. Sci Technol Wind. (2020) 18:247–8.

[6] Kong Y Zuo YL Li H. Construction and reliability and validity study of a satisfaction survey scale for community internships among general medical students. Chin Gen Pract. (2021) 24:3250–7.

[7] Xu F Zhang XH Ma R Bai H Zhang M Zhang JJ. Application of open experiments in experimental teaching of preventive medicine majors. J Kunming Med Univ. (2016) 37:144–7.39493181

[8] Fan CM Yin YM. Research on the construction of a practice teaching system centered on student satisfaction. Educ Teach Forum. (2020) 19:200–1.

[9] Xiao YN Li J. Study on the factors influencing students' satisfaction with practical teaching in universities. J Jishou Univ (Soc Sci Ed). (2018) 39:214–6.

[10] Chen ZQ Zhao D Luo XM Liu X Mao L. Problem-based teaching of health inspection experimental courses. Lab Res Explor. (2020) 39:192–194, 207.

[11] Zou ZH Yan WL Chen MX Wang M Zhou ZR Wu FH. Analysis of satisfaction among undergraduate students majoring in public health. Chin J Health Lab Technol. (2020) 30:3068–70.

